# Cortisol Directly Stimulates Spermatogonial Differentiation, Meiosis, and Spermiogenesis in Zebrafish (*Danio rerio*) Testicular Explants

**DOI:** 10.3390/biom10030429

**Published:** 2020-03-10

**Authors:** Aldo Tovo-Neto, Emanuel R. M. Martinez, Aline G. Melo, Lucas B. Doretto, Arno J. Butzge, Maira S. Rodrigues, Rafael T. Nakajima, Hamid R. Habibi, Rafael H. Nóbrega

**Affiliations:** 1 Aquaculture Program (CAUNESP), São Paulo State University, Jaboticabal, 14884-900 SP, Brazil; aldotovo@gmail.com (A.T.-N.); maira.bio2012@gmail.com (M.S.R.); 2 Department of Biological Sciences, University of Calgary, Calgary, T2N 1N4 AB, Canada; habibi@ucalgary.ca; 3 Reproductive and Molecular Biology Group, Department of Morphology, Institute of Biosciences, São Paulo State University, Botucatu, 18618-970 SP, Brazil; erm_martinez@yahoo.com.br (E.R.M.M.); alinegmelo16@gmail.com (A.G.M.); lucasbdoretto@gmail.com (L.B.D.); juliano.butzge@hotmail.com (A.J.B.); nakajimatakahiro.r@gmail.com (R.T.N.); 4 Department of Physiology and Pharmacology, University of Calgary, Calgary, T2N 1N4 AB, Canada

**Keywords:** cortisol, glucocorticoid receptor, spermatogenesis, zebrafish

## Abstract

Cortisol is the major endocrine factor mediating the inhibitory effects of stress on vertebrate reproduction. It is well known that cortisol affects reproduction by interacting with the hypothalamic–pituitary–gonads axis, leading to downstream inhibitory and stimulatory effects on gonads. However, the mechanisms are not fully understood. In this study, we provide novel data demonstrating the stimulatory effects of cortisol on spermatogenesis using an ex vivo organ culture system. The results revealed that cortisol treatment did not modulate basal androgen production, but it influenced transcript levels of a selected number of genes involved in the zebrafish testicular function *ar* (androgen receptor), *star* (steroidogenic acute regulatory), *cyp17a1* (17α-hydroxylase/17,20 lyase/17,20 desmolase), *cyp11a2* (cytochrome P450, family 11, subfamily A, polypeptide 2), *hsd11b2* (11-beta hydroxysteroid dehydrogenase), *cyp2k22* (cytochrome P450, family 2, subfamily K, polypeptide 22), *fkbp5* (FKBP prolyl isomerase 5), *grα* (glucocorticoid receptor alpha), and *grβ* (glucocorticoid receptor beta) in a short-term culture. We also showed that cortisol stimulates spermatogonial proliferation and differentiation in an androgen independent manner as well as promoting meiosis and spermiogenesis by increasing the number of spermatozoa in the testes. Moreover, we demonstrated that concomitant treatment with RU 486, a potent glucocorticoid receptor (Gr) antagonist, did not affect the cortisol effects on spermatogonial differentiation but blocked the induced effects on meiosis and spermiogenesis. Supporting the Gr-mediated effects, RU 486 nullified the cortisol-induced expression of *sycp3l* (synaptonemal complex protein 3), a marker for the meiotic prophase that encodes a component of the synaptonemal complex. This is consistent with in silico analysis that found 10 putative GREs (glucocorticoid response elements) upstream of the zebrafish *sycp3l*. Finally, we also showed that *grα* mRNA is expressed in Sertoli and Leydig cells, but also in several types of germ cells, including spermatogonia and spermatocytes. Altogether, this evidence indicates that cortisol exerts paracrine roles in the zebrafish testicular function and spermatogenesis, highlighting its effects on spermatogonial differentiation, meiosis, and spermiogenesis.

## 1. Introduction

Cortisol is the primary glucocorticoid hormone in teleost fish released in response to a stressor exposure upon the activation of the hypothalamic–pituitary–interrenal (HPI) axis (for a review see Schreck and Tort [1]; Milla et al. [2]; Faught and Vijayan [3]). The activation of the HPI axis first involves the release of corticotropin-releasing factor (CRF) from the hypothalamic preoptic area, which in turn stimulates the anterior pituitary to secrete the adrenocorticotropic hormone (ACTH) [4,5,6,7]. The circulating ACTH acts on the interrenal gland (analogous to the adrenal gland in mammals) and stimulates the synthesis and secretion of cortisol [3,5,8,9]. An important action of cortisol is to increase glucose bioavailability and improve an organism’s ability to cope with stress condition. In addition, glucocorticoids can also impact other physiological functions, including reproduction [1,3,5,8]. In this regard, it is widely accepted that stress and cortisol may have inhibitory effects on reproduction in vertebrates including teleost fish [3,10,11,12]. Most of the negative effects came from studies with females (especially in salmonid species), where it has been shown that cortisol suppresses the brain–pituitary–gonadal (BPG) axis, leading to a lower pituitary gonadotropin content, reduced plasma sex steroid levels, and decreased gonadal weight [13,14]. However, the effect of cortisol on testicular function and spermatogenesis has not been fully investigated in fish (for a review see Milla et al. [2]). In two different species of trout, stress-mediated cortisol elevation exerted deleterious effects on reproduction, and reduction of plasma androgen (testosterone and 11-Ketotestosterone (11-KT)) levels in sexually mature brown trout, *Salmo* trutta [15], and lower sperm quality in rainbow trout, *Oncorhynchus mykiss* [16]. In common carp, *Cyprinus carpio*, stress (temperature changes) and cortisol treatment resulted in reduced gonadosomatic index (GSI), delayed testicular development, and decreased plasma androgen levels [17,18]. Furthermore, chronically elevated cortisol levels in common carp affected all parts of the BPG axis by decreasing hypothalamic GnRH level; pituitary *fshβ* mRNA level and testicular androgen production in vitro [17,18,19]. In contrast, cortisol was found to increase the GSI and activated spermatogenesis in immature knifefish (*Notopterus notopterus*) [20]. In addition, cortisol (0.1–100 ng/mL) was found to directly stimulate DNA replication and spermatogonial proliferation in testicular fragments of immature Japanese eel, *Anguilla japonica* [21]. Moreover, higher concentration of cortisol (100 ng/mL) was found to stimulate testicular production of 11-KT in vitro, which suggests that cortisol-induced spermatogonial proliferation might be mediated by androgens in Japanese eel [21].

Studies using seasonal species (salmonids, goldfish, and common carp) have demonstrated increase in plasma cortisol level during pre-spawning and spawning periods in both mature male and female (see reviews Milla et al. [2]; Faught and Vijayan [3]). During vitellogenesis, cortisol incorporation into yolk is essential for proper progeny development, and during oocyte maturation, cortisol stimulates the production of maturing-inducing hormone (MIH; 17-alpha, 20-beta-dihydroxy-4-prenen-3-one), and oocyte sensitivity to MIH as well as being important for hydration and ovulation [3,22,23,24,25]. However little information is available on the role of cortisol in the regulation of testicular maturation in fish, in particular its potential role as a paracrine regulator of testicular function. In this context, it is noteworthy to mention that fish gonads of both sexes are able to produce cortisol, supporting the hypothesis that cortisol plays a role as paracrine regulator of testicular function [3,26,27,28,29,30,31,32,33].

The aim of this study was to investigate the direct action of cortisol on zebrafish (*Danio rerio*) testicular function using an ex vivo organ culture system established for this species [34]. We first evaluated the short-term effects of different concentrations of cortisol on 11-KT release and transcript abundance for a selected number of genes involved in testicular function. Subsequently, we investigated long-term effects of cortisol, in the presence or absence of a glucocorticoid receptor (Gr) antagonist, on zebrafish spermatogenesis by histomorphometrical, spermatogonial proliferation, and transcript measurement. To further support the transcript data, in silico analyses were performed to investigate glucocorticoid response elements (GREs) in the promoter regions of the differentially expressed genes affected by cortisol. Finally, we identified the cellular expression sites for the glucocorticoid receptors (Grs) in the zebrafish testes. Altogether these data clearly demonstrate that cortisol exerts a role in the regulation of zebrafish testicular function via Gr-expressing somatic and germ cells, as well as highlighting the stimulatory effects on zebrafish spermatogenesis, in particular on spermatogonial differentiation and meiosis.

## 2. Materials and Methods

### 2.1. Animals

Adult male of zebrafish (outbred) were bred and raised in the aquarium facility of Department of Morphology, Biosciences Institute, São Paulo State University. Handling and experimentation were consistent with Brazilian legislation regulated by National Council for the Control of Animal Experimental (CONCEA) and Ethical Principles in Animal Research (Protocol n. 671-CEUA).

### 2.2. Testis Tissue Culture

Testes were dissected out and cultured using an ex vivo organ culture system as previously described [34]. For short-term incubations (18 h for steroid release and gene expression analysis), testes were submerged in a culture medium, while for long-term exposures (7 days for morphology, BrdU assay, and gene expression analysis), testes were placed on a nitrocellulose membrane on top of a cylinder of agarose and cultivated with 1mL medium in 24-well flat-bottom plates, as previously described [34].

### 2.3. Short-Term (18 h) Incubation

After dissection, one testis was cultivated in the Lebovitz medium (L-15) (Sigma-Aldrich, St. Louis, USA), while its contra-lateral one in L-15 containing increasing levels of cortisol (0.1, 1, 10, 100, and 1000 ng/mL). For this experiment, eight adult males were used for each concentration, totaling 40 animals. Testes were incubated in 96-well plates containing 200 µL solution in each well at 28 °C. Following the period of incubation, testes were individually weighed and stored at −80 °C for gene expression analysis, and the medium was collected and stored at −20 °C for androgen release (11-Ketotestosterone, 11-KT) assay (see below).

### 2.4. In Vitro 11-Ketotestosterone (11-KT) Release

This technique was used to examine if different concentrations of cortisol (0.1, 1, 10, 100, and 1000 ng/mL) modulate basal androgen release by zebrafish testis. The androgen (11-KT) release capacity of zebrafish testicular tissue into culture medium was measured after a short-term (18 h) ex vivo culture system as described previously [35]. The levels of 11-KT released in the culture medium were quantified by an enzyme-linked immunosorbent assay (ELISA) with commercial kit (Cayman Chemical, Ann Arbor, USA) following manufacturer’s instructions.

### 2.5. Gene Expression Analysis by Real-Time, Quantitative PCR (qPCR)

Total RNA from testes (short-term and long-term incubations) was extracted by using the commercial RNAqueous®-Micro kit (Ambion, Austin, USA), according to manufacturer’s instructions. The cDNA synthesis was performed as usual procedures [35]. qPCR reactions were conducted using 10 µL 2× SYBR-Green Universal Master Mix, 2 µL of forward primer (9 mM), 2 µL of reverse primer (9 mM), 1 µL of DEPC water, and 5 µL of cDNA. The relative mRNA levels of *ar* (androgen receptor), *cyp17a1* (17α-hydroxylase/17,20 lyase/17,20 desmolase), *star* (steroidogenic acute regulatory), *grα* (glucocorticoid receptor alpha), *grβ* (glucocorticoid receptor beta), *11β-hsd* or *hsd11b2* (11-beta hydroxysteroid dehydrogenase), *igf3* (insulin-like growth factor 3), *pou5f3* (POU domain, class 5, transcription factor 3), *nanog* (nanog homeobox), *dazl* (deleted in azoospermia-like), *sycp3l* (synaptonemal complex protein 3), *shippo* (outer dense fiber of sperm tails 3B), *cyp11a2* (cytochrome P450, family 11, subfamily A, polypeptide 2), *cyp11c1* (cytochrome P450, family 11, subfamily C, polypeptide 1), *cyp2k22* (cytochrome P450, family 2, subfamily K, polypeptide 22), *fdx1b* (ferredoxin 1b), *fkbp5* (FKBP prolyl isomerase 5), *cyp19a1a* (cytochrome P450, family 19, subfamily A, polypeptide 1a), *amh* (anti-Mullerian hormone), and *dmrt1* (doublesex and mab-3 related transcription factor 1) were evaluated in the different experiments. The mRNA levels of the targets (Cts) were normalized by the reference genes *ef1* (elongation factor 1) and *β-actin*, expressed as relative values of control group (as fold induction), according to the 2^−(ΔΔCT)^ method. Primers were designed based on zebrafish sequences available at Genbank (NCBI, https://www.ncbi.nlm.nih.gov/genbank/) (Table 1).

### 2.6. Long-Term (7 Days) Incubation

In order to study the effects of cortisol on zebrafish spermatogenesis, three sets of experiments using long-term incubations were carried out to perform histomorphometrical analysis (*n* = 8), BrdU incorporation (*n* = 8), and gene expression (*n* = 8). After dissecting out the testes (paired structure), each testis (left and right) was placed on a nitrocellulose membrane measuring 0.25 cm^2^ (25 µm of thickness and 0.22 µm of porosity) on top of a cylinder of agarose (1.5% *w*/*v*, Ringer’s solution—pH 7.4) with 1 mL of culture medium into a 24-well plate. In this system, one testis (left) was incubated in the presence of cortisol (stressed levels: 100 ng/mL) and its contra-lateral one (right) in a basal culture medium (L-15). The medium was changed every 3 days of culture. After 7 days, testes were collected for histomorphometrical analysis and BrdU assay (see below). Testes were also collected for gene expression analysis, where total RNA was extracted from testis explants (*n* = 8) and the relative mRNA levels of *star*, *cyp17a1*, *cyp11a2*, *hsd11b2*, *cyp19a1a*, *cyp11c1*, *ar*, *grα*, *grβ*, *igf3*, *amh*, *nanog*, *pou5f3*, *dazl*, *sycp3l*, *shippo, dmrt1*, and *fkbp5* were evaluated as described above (qPCR).

### 2.7. Histomorphometrical Analysis

Zebrafish testicular explants were fixed in 4% buffered glutaraldehyde at 4 °C overnight, dehydrated, embedded in Technovit (7100-Heraeus Kulzer, Wehrheim, Germany), sectioned at 4µm thickness, and stained with 0.1% toluidine blue to quantify the different germ cell types at 40× and 100× objectives using a high-resolution light microscope (Leica DM6000 BD, Leica Microsystems, Wetzlar, Germany). For the morphometrical analysis, five histological fields for each animal (*n* = 8) were randomly selected for counting the frequency of germ cell cysts (type A undifferentiated and differentiated spermatogonia, type B spermatogonia, spermatocytes, and spermatids). The relative number of spermatozoa per area was estimated as described by Fallah and collaborators [43] using the histological images (40× objective) aligned with the optimized parameters (magnification, 8-bit image type). In this analysis, background subtraction, threshold adjustment, and watershed separation of particles that resulted in a black and white picture of the highlighted spermatozoa were used to count the relative number of spermatozoa in the control and treatment groups.

### 2.8. Germ Cell Proliferation—BrdU Incorporation

To evaluate the effects of cortisol on germ cell proliferation, 100 µg/mL bromodeoxyuridine (BrdU) was added to the culture medium during the last 6 h of incubation of the long-term culture. After incubation, zebrafish testes (*n* = 8) were fixed at 4 °C overnight in freshly prepared methacarn (60% [*v*/*v*] absolute ethanol, 30% chloroform, and 10% acetic acid) for 4 h. Following up, tissues were dehydrated, embedded in Technovit 7100 (7100-Heraeus Kulzer, Wehrheim, Germany), sectioned at 4µm thickness, and submitted to BrdU immunodetection, as previously described [40]. The mitotic index or BrdU incorporation ratio of type A undifferentiated, differentiated, and type B spermatogonia was performed as described previously [35,44].

### 2.9. Glucocorticoid Receptor Antagonist (RU-486)

To evaluate whether the cortisol effects were mediated by glucocorticoid receptor, a potent antagonist of glucocorticoid action in fish (RU-486) (see Goos and Consten [19]) was used in the long-term culture. For this purpose, zebrafish testes (*n* = 16) were incubated with cortisol in the presence or absence of RU-486 (1 µg/mL) for 7 days of culture. After the period of incubation, testes were collected for histomorphometrical analysis (*n* = 8) and gene expression (*n* = 8), as described above.

### 2.10. In Silico Analysis

To retrieved the putative glucocorticoid response element (GRE) sequences upstream of the target genes *sycp3l* (NM_001040350.1), *shippo* (NM_199958.1), *ar* (NM_001083123.1), *hsd11b2* (NM_212720.2), *star* (NM_131663.1), *cyp17a1* (NM_212806.3), and *gr* (NM_001020711.3), 19 GRE sequences described from the literature were used as references [45,46,47]. The flanking regions (2000 bp), 3′ and 5′, of the analyzed genes were extracted from National Center for Biotechnology Information (NCBI) and the GRE sequences were prospected. The predicted GRE sequences were filtered by identity, where at least eight of the 15 nucleotides were identical with the GRE consensus or GRE seabass sequence. Additionally, the TATA-Box, CAAT-Box, and GC-Box sequences were searched for each gene flanking region.

### 2.11. In Situ Hybridization

PCR product for *grα* (most expressed isoform) was generated with primers (Table 1). The specific PCR product was gel purified and served as a template for digoxigenin (DIG)-labelled cRNA probe synthesis using the RNA labeling (Roche, Basel, Switzerland) kit. The in situ hybridization was performed with adaptations of the Thisse and Thisse protocol [48]. Zebrafish testes were fixed overnight in 4% phosphate-buffered paraformaldehyde (pH 7.4) in RNase-free conditions. After fixation, testes were dehydrated, diaphanized, embedded in paraffin (Paraplast^®^, Sigma, St. Louis, USA), and sectioned with 5 μm thickness. The slides were rehydrated and washed with Tris buffer phosphate (PBT, pH: 7.4) and TrisHCl buffer (0.05 M, pH: 7.5). The material was treated with 20 μg/mL of proteinase K at 37 °C for 20 min and subsequently incubated overnight at 70 °C with the hybridization solution containing either sense or antisense RNA probe. In the following step, the slides were incubated with 1:2000 diluted primary anti-DIG-AP antibody (anti-digoxigenin-alkaline phosphatase conjugated) in the same blocking solution at 4 °C overnight. Slides were washed and incubated with a fluorescent detection set HNPP/Fast Red kit (Roche, Basel, Switzerland) according to the manufacturer’s instructions. The reaction was stopped by washing in 1× PBS. Sections were then counterstained with DAPI, slides were mounted with Fluorescent mounting medium (Dako, North America Inc, Carpinteria, CA, USA), and images were taken using the Leica DMI 4000B microscope (Leica Microsystems, Wetzlar, Germany).

### 2.12. Differential Platting

Testes (*n* = 10) were digested with 0.2% collagenase and 0.12% dispase, as described previously [35]. The total cell suspension was then submitted to a differential plating method, where the somatic cells adhere to the bottom of the plate, while germ cells either remain in suspension after 2–3 days of culture or only weakly associated with the firmly adhering somatic cells [49]. By using this method, germ and somatic cell enriched fractions can be obtained [49]. Total RNA from the cell suspensions (total, germ cell enriched, and somatic cell enriched) was extracted using RNAqueous®-Micro kit (Ambion, Austin, TX, USA), according to manufacturer’s instructions. After cDNA synthesis, the relative mRNA levels of the target genes (*grα* and *grβ*) were determined by qPCR as described above.

### 2.13. Statistical Analysis

Data are presented as mean ± SEM (standard error of mean). Significant differences were identified using Student’s *t*-test (paired or unpaired) (*p* < 0.05). Comparisons of more than two groups were performed with one-way ANOVA followed by Student–Newman–Keuls test (*p* < 0.05). All data analysis was performed by Graph Pad Prism software 4.0 (*Graph Pad Software*, Inc., San Diego, CA, USA, http://www.graphpad.com).

## 3. Results

### 3.1. Cortisol Does Not Modulate Basal Androgen Release but Influences Transcript Levels of a Selected Number of Genes

**Figure 1:**
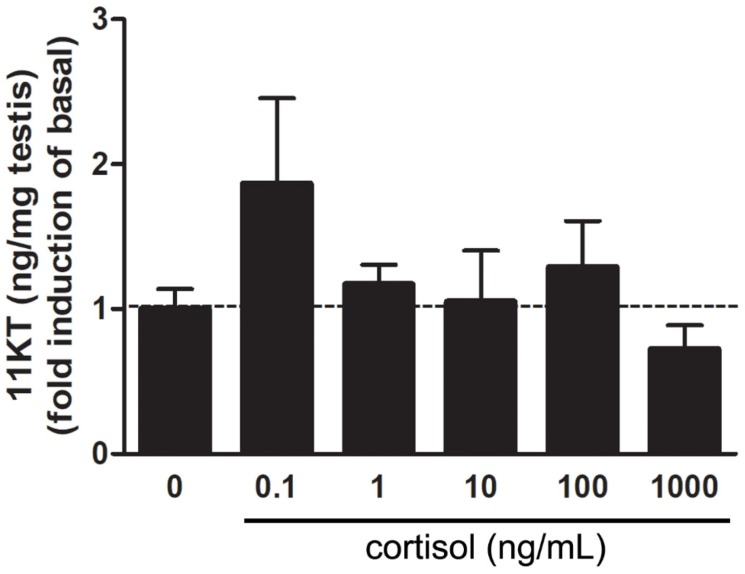
Androgen release from zebrafish testicular explants. Amounts of 11-KT (11-Ketotestosterone) (ng/mg of testis weight) measured in the incubation media after short-term exposure (18 h) to increasing concentrations of cortisol (0, 0.1, 1, 10, 100, and 1000 ng/mL). Data are expressed as mean ± SEM fold change (*n* = 8 per concentration), relative to the control condition, which is set at 1 (dashed line).

**Figure 2:**
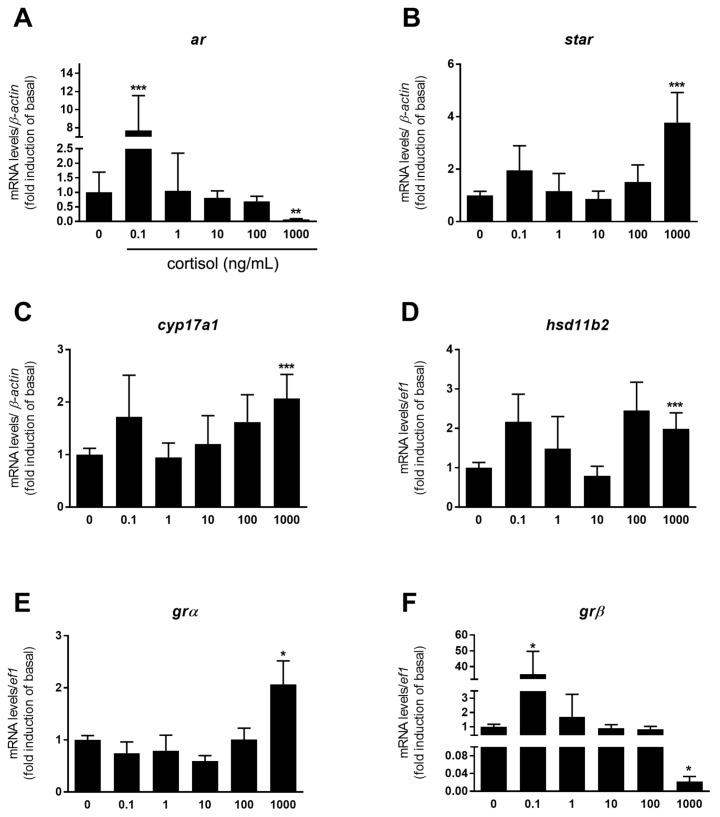
Relative mRNA levels of several selected genes in zebrafish testes incubated for 18 h (short-term exposure) to increasing concentrations of cortisol (0, 0.1, 1, 10, 100, and 1000 ng/mL). The selected target genes *ar* (androgen receptor) (**A**), *star* (steroidogenic acute regulatory) (**B**), *cyp17a1* (17α-hydroxylase/17,20 lyase/17,20 desmolase) (**C**), *hsd11b2* (11-beta hydroxysteroid dehydrogenase) (**D**), *grα* (glucocorticoid receptor alpha) (**E**), and *grβ* (glucocorticoid receptor beta) (**F**) were evaluated. Ct values were normalized with *β-actin* A–C (*ar*, *star*, *cyp17a1*) and *ef1* (elongation factor 1α) D–F (*hsd11b2*, *grα*, and *grβ*) and expressed as relative values of basal (0 ng/mL) levels of expression. Bars represent the mean ± SEM fold change (*n* = 8), relative to the control (basal, 0 ng/mL), which is set at 1. Paired *t*-test, *** *p* < 0.001; ** *p* < 0.01; * *p* < 0.05.

To evaluate whether cortisol modulates basal androgen release, zebrafish testes were incubated with different concentrations of cortisol (0.1, 1, 10, 100, and 1000 ng/mL) followed by measurement of 11-KT levels in the culture medium after 18h culture (Figure 1). Treatment with cortisol did not affect basal 11-KT release (Figure 1). However, short-term exposure to cortisol induced changes in zebrafish testicular transcript levels (Figure 2A–F). The effect of cortisol on androgen receptor (*ar*) mRNA level was biphasic. Cortisol increased basal *ar* mRNA levels about 8 fold at the lowest concentration tested (0.1 ng/mL), but decreased *ar* transcript level at the highest concentration of 1000 ng/mL (Figure 2A). Intermediary concentrations (1, 10, and 100 ng/mL) had no effects on *ar* mRNA level (Figure 2A).

We also measured mRNA levels of some steroidogenic enzymes, such as *star* (steroidogenic acute regulatory protein), *cyp17a1* (cytochrome P450 enzyme family), *cyp11a2* (principal side chain cleavage enzyme in adult steroidogenesis), and *cyp11c1* (enzyme involved in androgen and cortisol biosynthesis), and also for Fdx1b which is an essential electron-providing cofactor to steroidogenic enzymes related to glucocorticoid and androgen production (Cyp11a2 and Cyp11c1). Treatment with the highest concentration of cortisol significantly increased the transcript levels of *star* and *cyp17a1* (~4 and ~2 fold, respectively) (Figure 2B,C), while *cyp11a2* was downregulated in this concentration (Figure S1A). Moreover, cortisol (lowest and highest concentration) was without any effect on *cyp11c1* mRNA level (Figure S1B). Interestingly, the highest concentration of cortisol increased the transcript levels of *hsd11b2* (hydroxysteroid 11-beta dehydrogenase 2) (Figure 2D), which encodes 11β-hydroxysteroid dehydrogenase (11β-HSD), that is an enzyme involved in the androgen synthesis and cortisol inactivation into cortisone. Transcript level of *fdx1* remained unaltered in the cortisol treatment when compared to control (Figure S1D). Although there were changes in steroidogenic enzyme transcript levels, cortisol treatment had no effect on 11-KT release (Figure 1). This is in agreement with the transcript level of *cyp2k22*, an androgen-responsive gene, that remained below control levels in the presence of cortisol (Figure S1C). Treatment with the highest concentration of cortisol increased mRNA levels of *fkbp5* (glucocorticoid responsive gene) (Figure S1E) and *grα* (glucocorticoid receptor alpha), but reduced *grβ* transcripts (Figure 2E,F). Interestingly, the lowest concentration of cortisol (0.1 ng/mL) stimulated the *grβ* transcript level by about 35 fold, compared to the control (Figure 2F).

### 3.2. Effects of Cortisol on Zebrafish Spermatogenesis: Stimulatory Roles on Spermatogonial Differentiation, Meiosis, and Spermiogenesis

**Figure 3:**
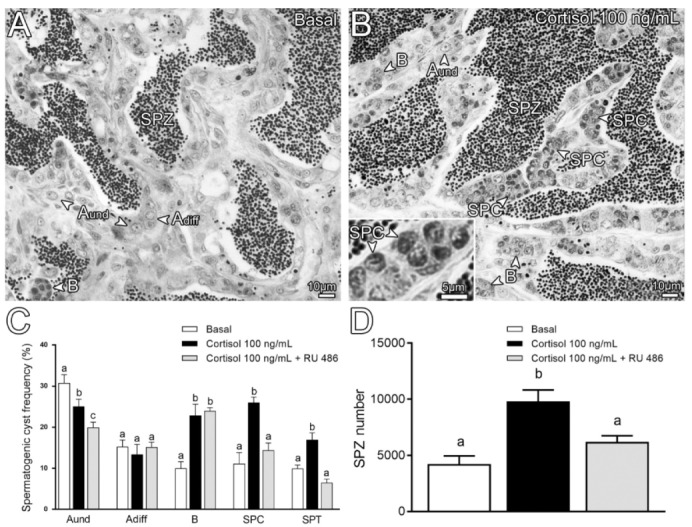
Histological sections from zebrafish testicular explants incubated for 7 days (long-term exposure) with 100 ng/mL cortisol compared with control (basal). (**A**) Basal (0 ng/mL). (**B**) Cortisol 100 ng/mL. Several germ cell types are indicated, such as type A undifferentiated spermatogonia (A_und_); type A differentiated spermatogonia (A_diff_); type B spermatogonia (B), spermatocytes (SPC), and spermatozoa (SPZ). (**C**) Histomorphometrical analysis of testicular explants incubated for 7 days with cortisol (100 ng/mL) in the absence or presence of 1 µg/mL RU 486 (glucocorticoid antagonist), compared to the control (basal). Bars (mean ± SEM; *n* = 8) represent the percentage of the spermatogenic cysts at different stages of germ cell development: A_und_; A_diff_; B; SPC and spermatids (SPT). (**D**) Spermatozoa quantification per field generated by using IMAGE J Software from zebrafish explants incubated for 7 days with either basal (L-15), cortisol (100 ng/mL), or cortisol (100 ng/mL) + RU 486 (1 µg/mL). ANOVA followed by Student–Newman–Keuls; different letters indicate significant differences (*p* < 0.05) between different treatment conditions.

In most of the teleost species, the circulating levels of cortisol is below 10 ng/mL, and increases by approximately 10 times during stress to about 100 ng/mL, which is similar to the values found in zebrafish and other fish species [2,50]. Based on that, we used the concentration of 100 ng/mL cortisol to study its long-term effects on zebrafish spermatogenesis using an ex vivo organ culture system. Morphological analysis revealed that cortisol (100 ng/mL) treatment stimulates zebrafish spermatogenesis (Figure 3A–D).

Testicular explants incubated with cortisol exhibited spermatogenic cysts at different stages of development and quantitatively larger number of spermatozoa compared to control (Figure 3A,B,D). Histomorphometrical analysis of spermatogenic cyst frequency revealed that cortisol treatment decreases type A undifferentiated spermatogonia (A_und_), but increases type B spermatogonia (Figure 3C). There was no change in type A differentiated spermatogonia (A_diff_) numbers (Figure 3C). Interestingly, the meiotic and post-meiotic cysts became more frequent in the cortisol treatment when compared to control (Figure 3B,C). Concomitant treatment with 1 µg/mL of RU 486, a potent glucocorticoid receptor antagonist (see review in Goos and Consten [19]) reversed the stimulatory effects of cortisol on zebrafish spermatogenesis (Figure 3C,D). Treatment with RU 486 was able to block the cortisol-induced meiotic and post-meiotic cysts (Figure 3C), and spermatozoa number (Figure 3D). However, RU 486 treatment was without effect on the cortisol induced changes in early stages of zebrafish spermatogenesis (spermatogonia type A_und_ and B) (Figure 3C). In our study, RU 486 treatment enhanced the inhibitory effect of cortisol on type A_und_ germ cell type (Figure 3C).

**Figure 4:**
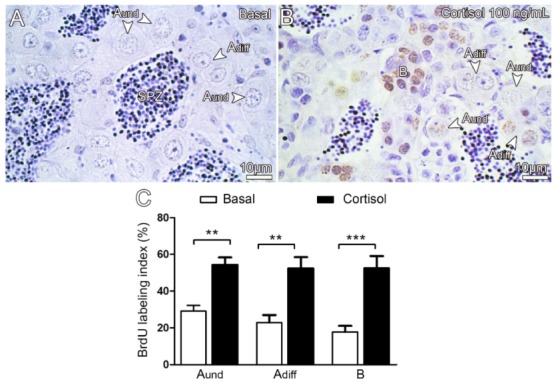
BrdU immunodetection from zebrafish testicular explants incubated for 7 days (long-term exposure) with 100 ng/mL cortisol compared with control (basal). (**A**) Basal. (**B**) Cortisol 100 ng/mL. Type A undifferentiated spermatogonia (A_und_); type A differentiated spermatogonia (A_diff_); type B spermatogonia (B) and spermatozoa (SPZ). (**C**) BrdU labeling index of A_und_, A_diff_, and B in zebrafish testis treated in the absence (basal) or presence of cortisol (100 ng/mL) for 7 days of culture. Bars represent the mean ± SEM (*n* = 8). Paired *t*-test, *** *p* < 0.001; ** *p* < 0.01.

We also examined if cortisol could stimulate spermatogonial proliferation by evaluating the BrdU-labeling index of different types of spermatogonia (Figure 4A–D). Treatment with cortisol significantly increased number of BrdU-labeled A_und_, A_diff_, and B cells in the zebrafish testis (Figure 4). Thus, BrdU-mitotic index confirmed that cortisol stimulated proliferation of different types of spermatogonia cells (Figure 4C).

**Figure 5:**
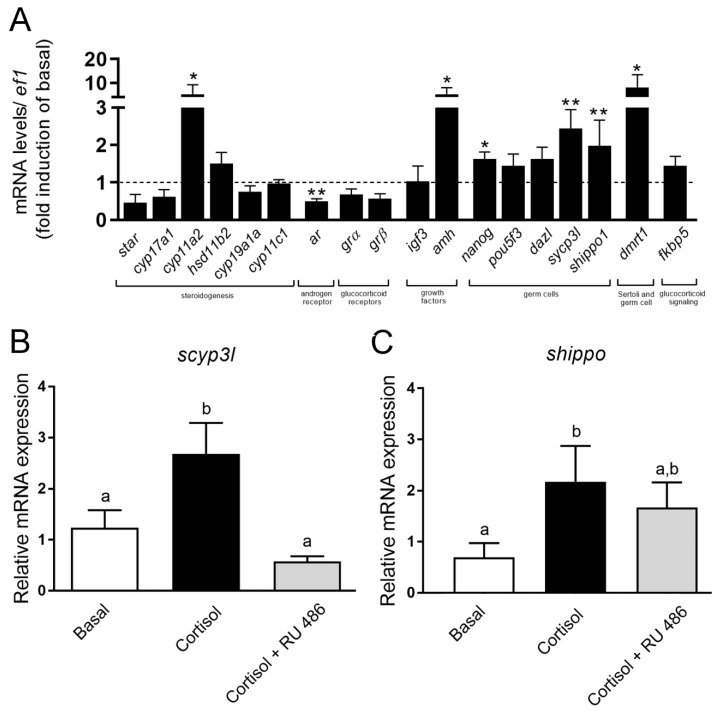
(**A**) Relative mRNA levels of several selected genes in zebrafish testes incubated for 7 days (long-term exposure) in the presence or absence of 100 ng/mL cortisol. The relative mRNA levels of *star* (steroidogenic acute regulatory), *cyp17a1* (17α-hydroxylase/17,20 lyase/17,20 desmolase), *cyp11a2* (cytochrome P450, family 11, subfamily A, polypeptide 2), *hsd11b2* (11-beta hydroxysteroid dehydrogenase), *cyp19a1a* (cytochrome P450, family 19, subfamily A, polypeptide 1a), *cyp11c1* (cytochrome P450, family 11, subfamily C, polypeptide 1), *ar* (androgen receptor), *grα* (glucocorticoid receptor apha), *grβ* (glucocorticoid receptor beta), *igf3* (insulin-like growth factor 3), *amh* (anti-Müllerian hormone), *nanog* (nanog homeobox), *pou5f3* (POU domain, class 5, transcription factor 3), *dazl* (deleted in azoospermia-like), *sycp3l* (synaptonemal complex protein 3), *shippo* (outer dense fiber of sperm tails 3B), *dmrt1* (doublesex and mab-3 related transcription factor 1), and *fkbp5* (FKBP prolyl isomerase 5) were normalized with the *ef1* (elongation factor 1α) levels. Bars represent the mean ± SEM fold change (*n* = 8), relative to the control (basal, 0 ng/mL), which is set at 1 (line). Paired *t*-test, ** *p* < 0.01; * *p* < 0.05. (**B**,**C**) Relative mRNA expression of *sycp3l* and *shippo* in the zebrafish testes incubated for 7 days with either basal (L-15), cortisol (100 ng/mL), or cortisol (100 ng/mL) + RU 486 (1 µg/mL). Expression levels of *sycp3l* and *shippo* were normalized with *ef1*. Bars represent the mean ± SEM (*n* = 8 per treatment). ANOVA followed by Student–Newman–Keuls; different letters indicate significant differences (*p* < 0.05) between treatment conditions.

The transcript abundance was found to be different for some selected genes in the zebrafish testicular explants exposed to cortisol (long-term) compared to control (Figure 5A). Transcript levels of steroidogenic enzymes, such as *star*, *cyp17a1*, *hsd11b2, cyp19a1a*, and *cyp11c1* remained unaltered in the cortisol treatment (Figure 5A). On the other hand, the principal side-chain cleavage enzyme in zebrafish steroidogenesis, *cyp11a2*, was significantly upregulated following cortisol exposure (Figure 5A). Interestingly, androgen receptor (*ar*) showed reduced mRNA levels in the zebrafish testicular explants exposed to cortisol when compared to control (Figure 5A). The transcript levels of glucocorticoid receptors (*grα* and *grβ*) and glucocorticoid responsive gene (*fkbp5*) were without changes in the cortisol treatment (Figure 5A). When evaluating the Sertoli cell growth factors, cortisol significantly increased the transcript level of *amh*, but was without effect on *igf3* mRNA levels (Figure 5A). Interestingly, *dmrt1* transcript levels in the zebrafish testes were increased following cortisol treatment (Figure 5A). In agreement with the histomorphometrical data, cortisol significantly increased the transcript levels of meiotic and post-meiotic genes, s*ycp3l* (synaptonemal complex, marker of primary spermatocytes) and *shippo* (also known as outer dense fiber 3 or ODF3, marker of spermatids), respectively, after 7 days culture (Figure 5A–C).

The transcript levels for genes related to spermatogonia, such as *pou5f*3 (undifferentiated spermatogonia) and *dazl* (differentiated spermatogonia), did not change following cortisol treatment (Figure 5A). On the other hand, mRNA levels of *nanog* (undifferentiated and differentiated spermatogonia) were increased in the zebrafish testes exposed to cortisol (Figure 5A). Treatment with RU 486 blocked cortisol-induced sc*yp3l* mRNA level (Figure 5B) but was without effect on *shippo* mRNA level (Figure 5C).

### 3.3. Several Putative Glucocorticoid Response Elements (GREs) were Found Upstream of Zebrafish Sycp3l

**Figure 6:**
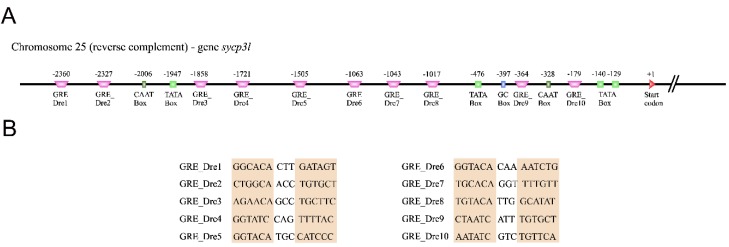
Schematic positions of predicted glucocorticoid response elements (GRE) upstream of the zebrafish *sycp3l* gene. (**A**) The nucleotide numbering was orientated according to the start codon (+1). Sequences of the *Danio rerio* GREs are indicated as Dre (purple trapeze). Green squares represent the TATA-Box; light green rectangles represent the CAAT-Box; blue rectangles represent the GC-Box. (**B**) Putative predicted GRE sequences (GRE_Dre1 to GRE_Dre10) located upstream of the zebrafish *sycp3l* gene, half 3′ and half 5′ of the GRE sequences were represented by salmon colored boxes.

To support our expression analysis, we also searched for putative glucocorticoid response elements (GREs) upstream of the differentially expressed genes in the cortisol treatment (Figure 6A,B, Table S1). The in silico analysis found several putative GREs in the seven analyzed genes, retrieving 47 different predicted sequences (GRE_Dre1–GRE_Dre47) (Table S1). In particular for the meiotic (*sycp3l*) gene, we strikingly found 10 putative GREs (GRE_Dre1–GRE_Dre10) upstream of the zebrafish *sycp3l* (Figure 6). With respect to the other genes, five putative GREs (GRE_Dre16–GRE_Dre20) were found upstream of the zebrafish *ar*, while five GREs (GRE_Dre23–GRE_Dre27) were predicted in the promoter region of *hsd11b2* (Table S1). The predicted GREs for the other differentially expressed genes are listed in Table S1.

### 3.4. Glucocorticoid Receptors are Expressed in Somatic (Sertoli and Leydig Cells) and Germ Cells of Zebrafish Testis

**Figure 7:**
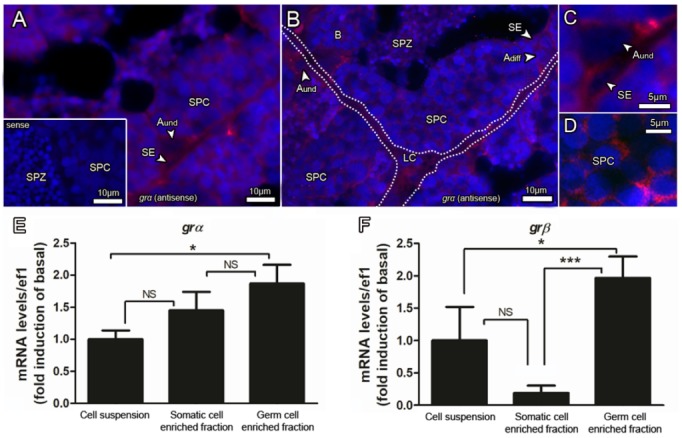
(**A**–**D**) Localization of *grα* (glucocorticoid receptor alpha) expressing cells in the zebrafish testis. Sections were subjected to in situ hybridization using antisense cRNA probe, followed by a fluorescent detection system (HNPP/Fast Red kit (Roche)). The red fluorescence indicates presence of signal, while nuclei were counterstained with DAPI (blue). No specific staining was observed when sections were incubated with sense cRNA probes (inset in (**A**)). *grα* mRNA was detected in Sertoli cells (SE), Leydig cells (LC), and several types of germ cells. Type A undifferentiated spermatogonia (A_und_); type A differentiated spermatogonia (A_diff_); type B spermatogonia (B), spermatocytes (SPC) and spermatozoa (SPZ). The dashed lines delimitate the interstitial compartment. *grα* (**E**) and *grβ* (**F**) in cell fractions (somatic and germ cell enriched cell fractions obtained from the differential plate method) compared with the total testicular cell suspension. ANOVA followed by Student–Newman–Keuls; *** *p* < 0.001; * *p* < 0.05; NS = not significant.

To identify the GRs expressing cells in the zebrafish testis, we performed in situ hybridization (ISH) using antisense and sense specific riboprobes (Figure 7A–D). Based on the shape and localization in the testis, *grα* mRNA was identified in Sertoli and Leydig cells, and also in several types of germ cells, in particular, type A_und_ and spermatocytes (Figure 7A–D). No specific signal was obtained when sections were incubated with the sense riboprobe (Figure 7A, inset). To support our data, *grα* and *grβ* transcripts were also evaluated in somatic and germ cell enriched fractions, which were obtained after the differential plating method, as previously described [49]. *grα* and *grβ* were detected in both somatic and germ cell enriched fractions (Figure 7E,F). While no differences were found for *grα* expression between somatic and germ cells, *grβ* appeared to be more expressed in the germ cells (Figure 7F). Interestingly, both transcripts (*grα* and *grβ*) exhibited higher expression in the germ cell enriched fraction when compared to the total cell suspension (Figure 7E,F). This observation supports a germ cell enrichment after the differential plating method (Figure 7E).

## 4. Discussion

The inhibitory effects of cortisol on fish spermatogenesis were demonstrated using in vivo experiments by incorporating cortisol into the feed, through hormone implants, and by associating the elevated plasma cortisol levels with changes in the fish testes [2,13,16,17,19,51]. To the best of our knowledge, this is the first demonstration of direct effects of cortisol on zebrafish adult testes. We provide novel data demonstrating stimulatory effects of cortisol on fish spermatogenesis. In the present study, we used low physiological (0.1 and 1 ng/mL), normal physiological (10 ng/mL), high physiological (100 ng/mL seen under stress condition), and supraphysiological (1000 ng/mL) concentrations, depending on the experiment.

We first evaluated dose-related direct action of cortisol on 11-KT production in the zebrafish testes in vitro. Treatment with cortisol was without effect on 11-KT release from the zebrafish testes. This is in agreement with the expression of *cyp2k22*, a well-established androgen-responsive gene in zebrafish [52,53], that remained below control levels in the presence of cortisol (0.1 and 1000 ng/mL). In this study, unfortunately, we did not measure steroids considered precursors of the steroidogenic pathway, such as 17α-hydroxyprogesterone, 11β-hydroxyandrostenedione, and 11β-hydroxytestosterone. Future studies measuring these steroids will add to our understanding of the mechanism involved in cortisol-mediated effects. In immature Japanese eel (*A. japonica*), cortisol (100 ng/mL cortisol) stimulated production of 11-KT from testicular fragments [21]. Similar findings were reported for pejerrey (*Odontesthes bonariensis*), where treatment with cortisol (0.1 and 1 ng/mL) also increased 11-KT release from adult testicular explants [54]. It was suggested that cortisol-induced androgen production in Japanese eel and pejerrey testes may be a by-product of cortisol inactivation by 11β-HSD [21,54,55]. In this context, 11β-HSD is responsible for converting cortisol into its inactive form cortisone, and in fish it is involved in the synthesis of 11-KT [21,54,55]. This implies a possible role for cortisol in the androgen biosynthesis by competition for the enzyme 11β-HSD. To support this hypothesis, it has been shown in pejerrey that cortisol (0.1 ng/mL) stimulated the expression of *hsd11b2*, whereas RU 486, which is a potent anti-glucocorticoid, blocked both cortisol-induced androgen production and *hsd11b2* expression [55]. In the present study, cortisol did not stimulate *hsd11b2* transcript level in zebrafish testes except for the highest dose tested (1000 ng/mL). Considering that 1000 ng/mL is a supraphysiological cortisol concentration [50], it is possible that higher *hsd11b2* transcript level observed may have been a response to metabolize the excess amount of cortisol into cortisone. Interestingly, in this case, the increase of *hsd11b2* did not result in higher 11-KT release by zebrafish testes, which might suggest that 11β-HSD has a preference for cortisol inactivation rather than androgen production in the zebrafish.

There is evidence for different regulatory mechanisms for *hsd11b2* in male and female zebrafish. Faught and collaborators [33] have shown that treatment of zebrafish follicles with 100 ng/mL cortisol (stressed levels) in vitro resulted in higher expression of *hsd11b2* (~7 times fold than control) after 4 h culture. In our work, we demonstrated that same concentration had no effect on testicular *hsd11b2* after 18 h and 7 days culture. This indicates that cortisol has more influence in the regulation of the ovarian *hsd11b2* rather than in the testicular one. The reason may be related to the function of 11β-HSD in the ovary which is essential to protect the oocytes from the abnormal levels of cortisol, therefore, restricting the excess of cortisol incorporation into eggs during maternal stress [3,33]. For zebrafish males, this buffering system to protect against high cortisol is not as important, detectable only at supraphysiological concentrations (1000 ng/mL).

We also examined if cortisol could modulate the expression of the selected testicular genes after 18h culture. Only the lowest and highest concentrations (0.1 and 1000 ng/mL) induced changes in transcript abundance following short-term incubation. In this regard, the lowest concentration of cortisol significantly increased *ar* mRNA levels, while the highest concentration suppressed this transcript in the zebrafish testis. The observed results were likely caused by direct action of cortisol on gene transcription, which is consistent with the demonstration of five putative GREs upstream of the zebrafish *ar* gene (GRE_Dre16–GRE_Dre20). The observed cortisol-induced changes in androgen receptor mRNA (*ar*) demonstrates that cortisol can modulate the androgen response in the zebrafish. Similar data was also reported in mammals, where glucocorticoids modulate androgen action by decreasing, in a dose dependent manner, the expression of both mRNA and protein levels of androgen receptor in a mammalian cell line [56]. There is evidence that cortisol may interact with androgen receptor (Ar), and it was shown that Ar has very low affinity for cortisol in the zebrafish testes (EC_50_ = 672 ng/mL) [57]. Therefore, it is possible that cortisol at the highest concentration tested (1000 ng/mL) may interact with androgen receptor signaling pathway in zebrafish. This interaction could be associated with the differential expression found for some steroidogenic enzymes, such as *star* and *cyp17a1* that were upregulated, whereas *cyp11a2* was significantly downregulated at this concentration.

The short-term exposure to cortisol also induced changes in the expression of glucocorticoid responsive gene (*fkbp5*) and glucocorticoid receptors (*grα* and *grβ*) in the zebrafish testicular explants. FK506 binding protein 5 (Fkbp5) is a glucocorticoid receptor chaperone protein, also known to be a robust glucocorticoid responsive gene in zebrafish [41,53,58]. This observation was confirmed in Fdx1b-deficient zebrafish, where males showed a decreased expression of *fkbp5* in response to the lower plasma cortisol levels [53]. In this study, only treatment with the highest concentration of cortisol increased *fkbp5* mRNA levels in the zebrafish testicular explants (short term incubation). This indicates that higher concentrations of cortisol are needed to induce *fkbp5* expression following short incubation. Treatment with cortisol also modulated the expression of glucocorticoid receptors, which is consistent with the demonstration of three putative GREs upstream of the zebrafish *gr* gene (GRE_Dre39–GRE_Dre41). Similar to humans, zebrafish express both *grα* and *grβ* isoforms which are splice variants derived from the Gr gene [59,60,61,62]. In the zebrafish testes, we found that *grα* is more expressed than *grβ* (*grα* Ct value = 23.9; *grβ* Ct value = 32.9). Interestingly, this pattern of expression seems to be similar to other tissues of zebrafish (spleen, liver, intestine, heart, brain, gills, and muscle) [61]. There is evidence that Grα interacts with glucocorticoids acting as a ligand-activated transcription factor regulating the expression of different target genes [60,61]. However, Grβ is unable to bind to glucocorticoids due to its shorter ligand-binding domain [60], and exerts a dominant-negative effect on *grα* transcriptional activity in humans [63] and zebrafish embryos [64]. In this regard, we demonstrate that *grα* and *grβ* were differentially regulated at the highest concentration of cortisol in the zebrafish testes; while *grα* mRNA levels were increased, *grβ* was strongly suppressed (~0.04-fold inhibition). This data could support the Grβ-mediated inhibition on *grα* transcription, in which the downregulation of *grβ* would be permissive for *grα* expression. For the other concentrations, we showed that neither basal nor stressed levels of cortisol induced *grα* or *grβ* expression in the zebrafish testis explants after 18h and 7 days culture. This data differs from studies using luciferase assays in COS-1 cells, where a lower concentration of cortisol (36 ng/mL) was able to induce zebrafish *grα* expression, while *grβ* mRNA levels remained unchanged in all concentrations tested [61]. With respect to *grβ*, our work showed that only the lowest concentration of cortisol (0.1 ng/mL) was able to stimulate the *grβ* transcription (~40-fold increase). Altogether, these results clearly indicate a differential regulation of *grα* and *grβ* by cortisol, and presumably, distinct roles for each isoform in the zebrafish testes. This topic deserves further studies to unravel the functions of the different Gr isoforms in the zebrafish testes.

In this study, we also examined the effects of cortisol (stressed levels—100ng/mL) in the zebrafish spermatogenesis. Previous studies have demonstrated that cortisol or stressful situations have negative impacts on spermatogenesis and testis development in fish [2,13,16,17,19,51], although it is not known if the observed effect was direct at the level of gonads or through inhibition of the brain–pituitary axis [17,19]. Moreover, there are several lines of evidence that cortisol is produced in the fish gonads, suggesting a possible paracrine role by cortisol in the regulation of gonadal function [2,26,27,28,29,30,31,32,33]. We strikingly found that cortisol stimulated zebrafish spermatogenesis by increasing the number of spermatozoa after 7 days of culture. Similar effects were observed in the immature knifefish, *Notopterus notopterus*, where the in vivo treatment with cortisol (40 and 60 µg/fish for 10 days) also promoted spermatogenesis [20]. To investigate the stimulatory effects of cortisol in zebrafish spermatogenesis, we first examined the cortisol actions on the spermatogonial phase. In this context, we showed that cortisol stimulated the mitosis of types A_und_, A_diff_, and B spermatogonia in the zebrafish testicular explants. In agreement, we showed increased transcript levels of *nanog* (transcription factor expressed in spermatogonia) and *dmrt1* (doublesex and mab-3 related transcription factor 1) in the zebrafish testicular explants following cortisol treatment (7 days). In zebrafish, Dmrt1 is expressed in both germ line and Sertoli cells [65]. Moreover, a recent study has demonstrated that Dmrt1 is required for proliferation and maintenance of male germ cells in zebrafish [66]. Altogether, these results support the effects of cortisol on inducing spermatogonial proliferation in the zebrafish testis. Since analysis on the proliferation activity does not provide information whether spermatogonia are heading for a self-renewal or a differentiation division, we also examined the frequency of these cell types in the zebrafish testis. Cortisol reduced the frequency of the most undifferentiated spermatogonia (type A_und_), while type A_diff_ remained unaltered, and type B spermatogonia (differentiated spermatogonia committed to meiosis) became more abundant in the testes. Based on the proliferation and frequency, we can assume that cortisol is favoring spermatogonial differentiation instead of self-renewal divisions of type A_und_. Similar effects on cell proliferation and differentiation have been linked to cortisol in other systems in humans and fish [67,68,69,70,71]. In zebrafish, in particular, it has been shown that cortisol promotes proliferation and epidermal ionocyte progenitor differentiation [72,73]. Interestingly, Ozaki and collaborators [21], using a similar organ culture system, also showed that cortisol (0.1–100 ng/mL) promoted spermatogonial proliferation in the immature testis of Japanese eel (*A. japonica*). In addition, the same study demonstrated that androgen (11-KT) production was stimulated when testicular fragments were incubated with stressed levels of cortisol [21]. Based on these results, Ozaki and collaborators [21] suggested that cortisol promoted spermatogonial proliferation by increasing 11-KT in the eel testis [21]. In the current study, stressed levels of cortisol did not affect the transcript levels for most steroidogenic enzymes (*star*, *cyp17a1*, *hsd11b2, cyp19a1a*, and *cyp11c1*) and had no effect on 11-KT production. However, mRNA levels of *cyp11a2,* which encodes a crucial enzyme for cholesterol side-chain cleavage in zebrafish steroidogenesis [74], were increased in the zebrafish testicular explants following cortisol treatment (7 days). Moreover, treatment with cortisol reduced the transcript levels of androgen receptor (*ar*) after 7 days of culture. In this regard, it has been shown that Ar-deficient zebrafish exhibited an increased expression of steroidogenic enzymes, such as *cyp11a2*, in their testes [75]. Similar pattern of transcript abundance has been reported in mice that lack androgen receptor (ARKO) [76]. Altogether, these data sustain the hypothesis that cortisol increased the transcript level of *cyp11a2* in response to the downregulation of androgen receptor in the zebrafish testicular explants.

We also examined whether cortisol-induced spermatogonial proliferation and differentiation could be mediated through a stimulatory growth factor, Igf3 (Insulin-like growth factor 3). Igf3 is a Sertoli cell derived growth factor that promotes spermatogonial proliferation and differentiation in the zebrafish testes [44,77,78,79]. Cortisol treatment did not affect *igf3* transcript level in the zebrafish testis. On the other hand, cortisol significantly increased the transcript level of *amh* (anti-Müllerian hormone) after 7 days of culture. Amh, a member of transforming growth factor β, is expressed in Sertoli cells [77,80], and exerts an important role in spermatogonial proliferation and differentiation, as reported in zebrafish and other fish species [80]. Moreover, studies have shown that Amh inhibits spermatogonial proliferation and differentiation in zebrafish testis [66,77,81]. Altogether, the increased levels of *amh* could be interpreted as a mechanism for protecting type A_und_ spermatogonia from excessive differentiation in response to cortisol. Taken together, we can conclude that cortisol acts through other mechanisms, rather than stimulating androgen and Igf3 signaling, to promote spermatogonial proliferation and differentiation in the zebrafish testis. In this context, transcriptomic analysis will be helpful to identify the cortisol target genes and pathways that mediate the stimulatory effects on spermatogonial proliferation and differentiation in the zebrafish testes.

When analyzing the effects on the other phases of spermatogenesis, we found that cortisol significantly increased the frequency of meiotic (spermatocytes) and post-meiotic (spermatids) cysts, which is consistent with the observed higher production of spermatozoa in the testes. These results were further confirmed by gene transcript analysis, where stressed levels of cortisol increased *sycp3l*, which is a marker for the meiotic prophase and encodes a component of the synaptonemal complex [82,83]. Furthermore, cortisol increased *shippo* mRNA levels, which is exclusively transcribed in early haploid cells [83,84]. Therefore, in addition to the spermatogonial proliferation and differentiation, cortisol also has stimulatory effects on meiosis and spermiogenesis of zebrafish. Cortisol also exerts stimulatory effects in oogenesis, including induction of oocyte meiotic maturation and ovulation, as reported in zebrafish and other fish species [2,3,33,85,86,87,88]. Taken together, these observations support the hypothesis that cortisol, as a local paracrine factor, exerts a spectrum of direct stimulatory actions on male and female gametogenesis. On the other hand, higher levels of circulating cortisol during stress response may suppress local stimulatory effects of cortisol by inhibiting GnRH and pituitary gonadotropin production [17,18,19].

To evaluate whether cortisol stimulatory effects were mediated by Gr, we used a potent Gr antagonist, RU 486. In the present study, RU 486 did not affect spermatogonial differentiation but blocked the cortisol effects on meiosis and spermiogenesis, suggesting that cortisol may act through different receptors at different stages of spermatogenesis. Cortisol action is mediated by two intracellular corticosteroid receptors; the Gr and the mineralocorticoid receptor (Mr) [72,73,87,88,89]. Considering that RU 486 did not block the formation of differentiated cells (pre-meiotic cells), we can speculate that cortisol role on zebrafish spermatogonial differentiation may be mediated by Mr. In contrast, a study using morpholinos in zebrafish showed that cortisol-driven ionocyte differentiation is mediated exclusively by Gr [73]. Interestingly, the same study demonstrated that epidermal stem cells of zebrafish, although not being affected by cortisol, were reduced in the Gr knockdown morphants [73]. In our study, the Gr antagonist also decreased the frequency of type A_und_, which contains the spermatogonial stem cell population in the zebrafish testes [35]. Similar to the epidermal stem cells [73], the observed reduction suggests that Gr signaling may be important for the survival of type A_und_. With respect to the late stages of zebrafish spermatogenesis, our results indicated that cortisol action on meiosis and spermiogenesis were mediated by Gr. In this regard, we showed that RU 486 strongly suppressed the cortisol-induced *sycp3l* expression in the zebrafish testes, while no differences were detected for *shippo* mRNA levels. To further investigate Gr-mediated effect on *sycp3l* expression, using in silico analysis, we identified 10 putative GREs upstream of the zebrafish *sycp3l* gene (GRE_Dre1–GRE_Dre10). Altogether, these observations indicate that cortisol promotes meiosis by increasing *sycp3l* expression in the zebrafish testes. In spite of not preventing *shippo*, RU 486 blocked cortisol effects on post-meiotic cysts and spermatozoa number, which is also evidence supporting involvement of Gr.

The in situ hybridization of zebrafish testis revealed that *grα* mRNA (the most abundant transcript) is present in Sertoli and Leydig cells, as well as in several types of germ cells, including spermatogonia and spermatocytes. We also demonstrated here that *grα* and *grβ* were expressed in somatic and germ cell enriched fractions, although *grβ* was more expressed in the germ cell fraction. In adult rats, GR was found in the interstitial cells (Leydig cells, macrophages, fibroblast, endothelial cells of blood vessels), peritubular myoid cells, Sertoli cells, spermatogonia, zygotene, and early pachytene primary spermatocytes [90,91,92,93]. Moreover, when disrupting GR expression in Sertoli cells of mice, there is a significant reduction of Sertoli cells, and a decreased number of meiotic and post-meiotic cells [94]. In fish, the only information about the Gr expressing cell types in testis comes from common carp, a closely related species to zebrafish [95]. In common carp, *gr* mRNA was expressed in several types of germ cells, from late spermatogonia to spermatocytes [95]. Interestingly, the most intense staining for Gr was found in meiotic cells [95]. No staining was observed in post-meiotic cells, and it was not possible to determine *gr* transcripts in Sertoli and Leydig cells [95]. Altogether, this evidence is consistent with the role of cortisol in zebrafish spermatogenesis; cortisol may act on Gr-expressing somatic cells (Sertoli and Leydig cells), as well as germ cells, where the most remarkable Gr-mediated effects is in primary spermatocytes, stimulating *sycp3l* mRNA levels and meiosis.

## 5. Conclusion

This study demonstrates for the first time the direct actions of cortisol on the zebrafish spermatogenesis. Cortisol stimulates spermatogonial proliferation and differentiation in an androgen independent manner as well as promoting meiosis and spermiogenesis by increasing the number of spermatozoa in the testes. While the cortisol actions in the early phases of spermatogenesis may be mediated by Mr, the effects on meiosis and spermiogenesis are dependent of Gr. With respect to the Gr-mediated effects, we clearly demonstrated that cortisol induces *sycp3l* mRNA levels. To support this evidence, 10 putative GREs were identified upstream of the zebrafish *sycp3l* gene. We also demonstrated that Gr is ubiquitously expressed in the zebrafish testes, being present in both somatic (Sertoli and Leydig cells) and germ cells. Future approaches, such as RNAseq, effects of Gr and Mr knockdown/knockout, localization of the production sites of cortisol in the testis, effects of cortisol-induced paternal stress in the spermatozoa, among others, would be necessary to unravel the direct and indirect effects of cortisol on fish testicular function.

## Figures and Tables

**Table 1:** Primers used for gene expression studies (qPCR) and to generate DNA templates for digoxigenin (DIG)-labeled cRNA probe syntheses for in situ hybridization (ISH).

Target	Primer Sequences (5′–3′)	Reference
*ef1α*	GCCGTCCCACCGACAAG (Fw) CCACACGACCCACAGGTACAG (Rv)	Morais et al. [36]
*b-actin*	AGACATCAGGGAGTGATGGT (Fw) CAATACCGTGCTCAATGGGG (Rv)	This paper
*ar*	ACGTGCCTGGCGTGAAAA (Fw) CAAACCTGCCATCCGTGAAC (Rv)	Morais et al. [36]
*star*	CCTGGAATGCCTGAGCAGAA (Fw) ATCTGCACTTGGTCGCATGAC (Rv)	Morais et al. [36]
*cyp17a1*	GGGAGGCCACGGACTGTTA (Fw) CCATGTGGAACTGTAGTCAGCAA (Rv)	Morais et al. [36]
*hsd11b2*	GGGGGTCAAAGTTTCCACTA (Fw) TGGAAGAGCTCCTTGGTCTC (Rv)	Tokarz et al. [37]
*grα*	ACTCCATGCACGACTTGGTG (Fw) GCATTTCGGGAAACTCCACG (Rv)	Manuel et al. [38]
*grβ*	GATGAACTACGAATGTCTTA (Fw) GCAACAGACAGCCAGACAGCTCACT (Rv)	Manuel et al. [38]
*sycp3l*	AGAAGCTGACCCAAGATCATTCC (Fw) AGCTTCAGTTGCTGGCGAAA (Rv)	García-Lopez et al. [39]
*dazl*	AGTGCAGACTTTGCTAACCCTTATGTA (Fw) GTCCACTGCTCCAAGTTGCTCT (Rv)	Morais et al. [36]
*nanog*	TGTCCTACAACAAGACTGAGCC (Fw) CAGGAATCTGGCGTGTGGG (Rv)	This paper
*shippo*	GATGCCTGGAGACATGACCAA (Fw) CAAAGGAGAAGCTGGGAGCTTT (Rv)	Leal et al. [40]
*igf3*	TGTGCGGAGACAGAGGCTTT (Fw) CGCCGCACTTTCTTGGATT (Rv)	Morais et al. [36]
*pou5f3*	GAGAGATGTAGTGCGTGTAT (Fw) GCTCGTAATACTGTGCTTCA (Rv)	This paper
*amh*	CTCTGACCTTGATGAGCCTCATTT (Fw) GGATGTCCCTTAAGAACTTTTGCA (Rv)	García-López et al. [39]
*cyp11a2*	ATACACTGGTGTGCTGGCAA (Fw) TATAGCCGTCGTGTCCACTC (Rv)	This paper
*cyp19a1*	AGATGTCGAGTTAAAGATCCTGCA (Fw) TCTACGTTTTCACCCGGTCG (Rv)	This paper
*cyp2k22*	TGCACTGTCAAACCTACGAG (Fw) CCTCCAAACCTCTCAATTTCCTC (Rv)	This paper
*dmrt1*	TGCCCAGGTGGCGTTACGG (Fw) CGGGTGATGGCGGTCCTGAG (Rv)	Griffin et al. [41]
*fkbp5*	TTCCACACTCGTGTTCGAGA (Fw) ACGATCCCACCATCTTCTGT (Rv)	Griffin et al. [41]
*fdx1b*	GAGCAGCGTATTTGTCACAGA (Fw) ACCATTGGCTCCAGTTTGTCA (Rv)	Griffin et al. [41]
*cyp11c1*	CCTCGGGCCCATATACAGAGA (Fw) CGTCCCGTTCTTGAGGAAGA (Rv)	Sreenivasan et al. [42]
*grα*	[T7Rpps]-CATTTCGGGAAACTCCACG (Fw) [T3Rpps]-ACTCCATGCACGACTTGGTG (Rv)	This paper

Fw, forward; Rv, reverse; T7Rpps—T7 RNA polymerase promoter sequence at its 5′-end (5′ CCGGGGGGTGTAATACGACTCACTATAGGG-3′), T3Rpps—T3 RNA polymerase promoter sequence at its 5′-end (T3′GGGCGGGTGTTATTAACCCTCACTAAAGGG-3′).

## Data Availability

The datasets generated during and/or analyzed during the current study are available from the corresponding author on reasonable request.

## Supplementary Materials

The following are available online at https://www.mdpi.com/2218-273X/10/3/429/s1, Figure S1. Relative mRNA levels of several selected genes in zebrafish testes incubated for 18 h (short-term exposure) to increasing concentrations of cortisol, Table S1. Identification of predicted glucocorticoid response elements (GRE) upstream of the target genes.

## References

[1] Schreck C.B., Tort L., Schreck C.B., Tort L., Farrell A., Braunner C. (2016). The Concept of Stress in Fish. Biology of Stress in Fish: Fish Physiology.

[2] Milla S., Wang N., Mandiki S.N.M., Kestemont P. (2009). Corticosteroids: Friends or foes of teleost fish reproduction?. Comp. Biochem. Physiol.-A Mol. Integr. Physiol..

[3] Faught E., Vijayan M.M. (2018). Maternal stress and fish reproduction: The role of cortisol revisited. Fish Fish..

[4] Schreck C.B., Pickering A.D. (1981). Stress and compensation in teleostean fishes: Response to social and physical factors. Stress and fish.

[5] Wendelaar Bonga S.E. (1997). The stress response in fish. Physiol. Rev..

[6] Barton B.A. (2002). Stress in Fishes: A Diversity of Responses with Particular Reference to Changes in Circulating Corticosteroids. Integr. Comp. Biol..

[7] Ramsay J.M., Feist G.W., Varga Z.M., Westerfield M., Kent M.L., Schreck C.B. (2006). Whole-body cortisol is an indicator of crowding stress in adult zebrafish, *Danio rerio*. Aquaculture.

[8] Mommsen T.P., Vijayan M.M., Moon T.W. (1999). Cortisol in teleosts: Dynamics, mechanisms of action, and metabolic regulation. Rev. Fish Biol. Fish..

[9] Dores R.M., Garcia Y. (2015). Views on the co-evolution of the melanocortin-2 receptor, MRAPs, and the hypothalamus/pituitary/adrenal-interrenal axis. Mol. Cell. Endocrinol..

[10] Pankhurst N.W., Van der Kraak G. (2000). Evidence that acute stress inhibits ovarian steroidogenesis in rainbow trout In Vivo, through the action of cortisol. Gen. Comp. Endocrinol..

[11] Pankhurst N.W., Schreck C.B., Tort L., Farrell A., Brauner C. (2016). Reproduction and Development. Biology of Stress in Fish: Fish Physiology.

[12] Schreck C.B., Contreras-Sanchez W., Fitzpatrick M.S. (2001). Effects of stress on fish reproduction, gamete quality, and progeny. Aquaculture.

[13] Carragher J.F., Sumpter J.P., Pottinger T.G., Pickering A.D. (1989). The deleterious effects of cortisol implantation on reproductive function in two species of trout, *Salmo trutta* L. and *Salmo gairdneri* Richardson. Gen. Comp. Endocrinol..

[14] Carragher J.F., Sumpter J.P. (1990). The effect of cortisol on the secretion of sex steroids from cultured ovarian follicles of rainbow trout. Gen. Comp. Endocrinol..

[15] Pickering A.D., Pottinger T.G., Carragher J., Sumpter J.P. (1987). The effects of acute and chronic stress on the levels of reproductive hormones in the plasma of mature male brown trout, *Salmo trutta* L.. Gen. Comp. Endocrinol..

[16] Campbell P.M., Pottinger T.G., Sumpter J.P. (1992). Stress Reduces the Quality of Gametes Produced by Rainbow Trout. Biol. Reprod..

[17] Consten D., Lambert J.G.D., Goos H.J.T. (2001). Cortisol affects testicular development in male common carp, *Cyprinus carpio* L., but not via an effect on LH secretion. Comp. Biochem. Physiol.-B Biochem. Mol. Biol..

[18] Consten D., Keuning E.D., Terlou M., Lambert J.G.D., Goos H.J.T. (2001). Cortisol effects on the testicular androgen synthesizing capacity in common carp, *Cyprinus carpio* L.. Fish Physiol. Biochem..

[19] Goos H.J.T., Consten D. (2002). Stress adaptation, cortisol and pubertal development in the male common carp, *Cyprinus carpio*. Mol. Cell. Endocrinol..

[20] Shankar D.S., Kulkarni R.S. (2000). Effect of cortisol on testis of freshwater fish *Notopterus notopterus* (Pallas). Indian J. Exp. Biol..

[21] Ozaki Y., Higuchi M., Miura C., Yamaguchi S., Tozawa Y., Miura T. (2006). Roles of 11β-Hydroxysteroid Dehydrogenase in Fish Spermatogenesis. Endocrinology.

[22] Hirose K. (1973). Biological Study on Ovulation in vitro of Fish-VI Effects of Metopirone (su-4885) on Salmon Gonadotropin- and Cortisol-induced in Vitro Ovulation in Oryzias Latipes. Bull. Japanese Soc. Sci. Fish..

[23] Jalabert B., Fostier A., Marcuzzi O., Heydorff M. (1984). The modulatory effect *in vitro* of oestradiol-17β, testosterone or cortisol on the output of 17α-hydroxy-20β-dihydroprogesterone by rainbow trout (*Salmo gairdneri*) ovarian follicles stimulated by the maturational gonadotropin s-GtH. Reprod. Nutr. Développement.

[24] Barry T.P., Riebe J.D., Parrish J.J., Malison J.A. (1997). Effects of 17α,20β-dihydroxy-4-pregnen-3-one on cortisol production by rainbow trout interrenal tissue in vitro. Gen. Comp. Endocrinol..

[25] Milla S., Jalabert B., Rime H., Prunet P., Bobe J. (2006). Hydration of rainbow trout oocyte during meiotic maturation and *in vitro* regulation by 17,20β-dihydroxy-4-pregnen-3-one and cortisol. J. Exp. Biol..

[26] Colombo L., Bern H.A., Pieprzyk J., Johnson D.W. (1973). Biosynthesis of 11-deoxycorticosteroids by teleost ovaries and discussion of their possible role in oocyte maturation and ovulation. Gen. Comp. Endocrinol..

[27] Goswami S.V., Lamba V.J., Sundararaj B.I. (1985). Gonadotrophin-induced oocyte maturation in the catfish, *Heteropneustes fossilis* (Bloch), requires steroidogenesis in both interrenal and ovary. Gen. Comp. Endocrinol..

[28] Canario A.V.M., Scott A.P. (1990). Identification of, and development of radioimmunoassays for 17α,21-dihydroxy-4-pregnene-3,20-dione and 3α,17α,21-trihydroxy-5β-pregnan-20-one in the ovaries of mature plaice (*Pleuronectes platessa*). Gen. Comp. Endocrinol..

[29] Scott A.P., Sherwood N.M., Canario A.V.M., Warby C.M. (1991). Identification of free and conjugated steroids, including cortisol and 17α,20β-dihydroxy-4-pregnen-3-one, in the milt of Pacific herring, *Clupea harengus pallasi*. Can. J. Zool..

[30] Scott A.P., Canario A.V.M., Sherwood N.M., Warby C.M. (1991). Levels of steroids, including cortisol and 17α,20β-dihydroxy-4-pregnen-3-one, in plasma, seminal fluid, and urine of Pacific herring (*Clupea harengus pallasi*) and North Sea plaice (*Pleuronectes platessa* L.). Can. J. Zool..

[31] Kime D.E., Scott A.P., Canario A.V. (1992). *In vitro* biosynthesis of steroids, including 11-deoxycortisol and 5 alpha-pregnane-3 beta,7 alpha,17,20 beta-tetrol, by ovaries of the goldfish *Carassius auratus* during the stage of oocyte final maturation. Gen. Comp. Endocrinol..

[32] Alsop D., Ings J.S., Vijayan M.M. (2009). Adrenocorticotropic hormone suppresses gonadotropin-stimulated estradiol release from zebrafish ovarian follicles. PLoS ONE.

[33] Faught E., Best C., Vijayan M.M. (2016). Maternal stress-associated cortisol stimulation may protect embryos from cortisol excess in zebrafish. R. Soc. Open Sci..

[34] Leal M.C., de Waal P.P., García-López Á., Chen S.X., Bogerd J., Schulz R.W. (2009). Zebrafish primary testis tissue culture: An approach to study testis function ex vivo. Gen. Comp. Endocrinol..

[35] Nóbrega R.H., Greebe C.D., van de Kant H., Bogerd J., França L.R., Schulz R.W. (2010). Spermatogonial Stem Cell Niche and Spermatogonial Stem Cell Transplantation in Zebrafish. PLoS ONE.

[36] Morais R.D.V.S., Nóbrega R.H., Gómez-González N.E., Schmidt R., Bogerd J., França L.R., Schulz R.W. (2013). Thyroid hormone stimulates the proliferation of sertoli cells and single type A spermatogonia in adult zebrafish (*Danio rerio*) testis. Endocrinology.

[37] Tokarz J., Norton W., Möller G., de Angelis M.H., Adamski J. (2013). Zebrafish 20β-Hydroxysteroid Dehydrogenase Type 2 Is Important for Glucocorticoid Catabolism in Stress Response. PLoS ONE.

[38] Manuel R., Gorissen M., Roca C.P., Zethof J., van de Vis H., Flik G., van den Bos R. (2014). Inhibitory avoidance learning in zebrafish (*Danio Rerio*): Effects of shock intensity and unraveling differences in task performance. Zebrafish.

[39] García-López A., de Jonge H., Nóbrega R.H., de Waal P.P., van Dijk W., Hemrika W., Taranger G.L., Bogerd J., Schulz R.W. (2010). Studies in Zebrafish Reveal Unusual Cellular Expression Patterns of Gonadotropin Receptor Messenger Ribonucleic Acids in the Testis and Unexpected Functional Differentiation of the Gonadotropins. Endocrinology.

[40] Leal M.C., Cardoso E.R., Nóbrega R.H., Batlouni S.R., Bogerd J., França L.R., Schulz R.W. (2009). Histological and Stereological Evaluation of Zebrafish (*Danio rerio*) Spermatogenesis with an Emphasis on Spermatogonial Generations. Biol. Reprod..

[41] Griffin A., Parajes S., Weger M., Zaucker A., Taylor A.E., O’Neil D.M., Müller F., Krone N. (2016). Ferredoxin 1b (Fdx1b) Is the Essential Mitochondrial Redox Partner for Cortisol Biosynthesis in Zebrafish. Endocrinology.

[42] Sreenivasan R., Jiang J., Wang X., Bártfai R., Kwan H.Y., Christoffels A., Orbán L. (2014). Gonad differentiation in zebrafish is regulated by the canonical Wnt signaling pathway. Biol. Reprod..

[43] Fallah H.P., Tovo-Neto A., Yeung E.C., Nóbrega R.H., Habibi H.R. (2019). Paracrine/autocrine control of spermatogenesis by gonadotropin-inhibitory hormone. Mol. Cell. Endocrinol..

[44] Nóbrega R.H., Morais R.D.V.S., Crespo D., de Waal P.P., França L.R., Schulz R.W., Bogerd J. (2015). Fsh stimulates spermatogonial proliferation and differentiation in zebrafish via Igf3. Endocrinology.

[45] Danesch U., Gloss B., Schmid W., Schütz G., Schüle R., Renkawitz R. (1987). Glucocorticoid induction of the rat tryptophan oxygenase gene is mediated by two widely separated glucocorticoid-responsive elements. EMBO J..

[46] Cornett L.E., Hiller F.C., Jacobi S.E., Cao W., McGraw D.W. (1998). Identification of a glucocorticoid response element in the rat β2- adrenergic receptor gene. Mol. Pharmacol..

[47] Ma P., Reddy K.P., Chan W.K., Lam T.J. (2004). Hormonal influence on amylase gene expression during Seabass (*Lates calcarifer*) larval development. Gen. Comp. Endocrinol..

[48] Thisse C., Thisse B. (2008). High-resolution in situ hybridization to whole-mount zebrafish embryos. Nat. Protoc..

[49] Hinfray N., Nóbrega R.H., Caulier M., Baudiffier D., Maillot-Maréchal E., Chadili E., Palluel O., Porcher J.M., Schulz R., Brion F. (2013). Cyp17a1 and cyp19a1 in the zebrafish testis are differentially affected by oestradiol. J. Endocrinol..

[50] Tea J., Alderman S.L., Gilmour K.M. (2019). Social stress increases plasma cortisol and reduces forebrain cell proliferation in subordinate male zebrafish (*Danio rerio*). J. Exp. Biol..

[51] Milla S., Terrien X., Sturm A., Ibrahim F., Giton F., Fiet J., Prunet P., Le Gac F. (2008). Plasma 11-deoxycorticosterone (DOC) and mineralocorticoid receptor testicular expression during rainbow trout *Oncorhynchus mykiss* spermiation: Implication with 17alpha, 20beta-dihydroxyprogesterone on the milt fluidity?. Reprod. Biol. Endocrinol..

[52] Fetter E., Smetanová S., Baldauf L., Lidzba A., Altenburger R., Schüttler A., Scholz S. (2015). Identification and Characterization of Androgen-Responsive Genes in Zebrafish Embryos. Environ. Sci. Technol..

[53] Oakes J.A., Li N., Wistow B.R.C., Griffin A., Barnard L., Storbeck K.-H., Cunliffe V.T., Krone N.P. (2019). Ferredoxin 1b Deficiency Leads to Testis Disorganization, Impaired Spermatogenesis, and Feminization in Zebrafish. Endocrinology.

[54] Fernandino J.I., Hattori R.S., Kishii A., Strüssmann C.A., Somoza G.M. (2012). The Cortisol and Androgen Pathways Cross Talk in High Temperature-Induced Masculinization: The 11β-Hydroxysteroid Dehydrogenase as a Key Enzyme. Endocrinology.

[55] Fernandino J.I., Hattori R.S., Acosta O.D.M., Strüssmann C.A., Somoza G.M. (2013). Environmental stress-induced testis differentiation: Androgen as a by-product of cortisol inactivation. Gen. Comp. Endocrinol..

[56] Burnstein K.L., Maiorino C.A., Dai J.L., Cameron D.J. (1995). Androgen and glucocorticoid regulation of androgen receptor cDNA expression. Mol. Cell. Endocrinol..

[57] de Waal P.P., Wang D.S., Nijenhuis W.A., Schulz R.W., Bogerd J. (2008). Functional characterization and expression analysis of the androgen receptor in zebrafish (*Danio rerio*) testis. Reproduction.

[58] Eachus H., Zaucker A., Oakes J.A., Griffin A., Weger M., Güran T., Taylor A., Harris A., Greenfield A., Quanson J.L. (2017). Genetic Disruption of 21-Hydroxylase in Zebrafish Causes Interrenal Hyperplasia. Endocrinology.

[59] Hollenberg S.M., Weinberger C., Ong E.S., Cerelli G., Oro A., Lebo R., Brad Thompson E., Rosenfeld M.G., Evans R.M. (1985). Primary structure and expression of a functional human glucocorticoid receptor cDNA. Nature.

[60] Encío I.J., Detera-Wadleigh S.D. (1991). The genomic structure of the human glucocorticoid receptor. J. Biol. Chem..

[61] Schaaf M.J.M., Champagne D., van Laanen I.H.C., van Wijk D.C.W.A., Meijer A.H., Meijer O.C., Spaink H.P., Richardson M.K. (2008). Discovery of a Functional Glucocorticoid Receptor β-Isoform in Zebrafish. Endocrinology.

[62] Alsop D., Vijayan M. (2009). The zebrafish stress axis: Molecular fallout from the teleost-specific genome duplication event. Gen. Comp. Endocrinol..

[63] Oakleyt R.H., Jewell C.M., Yudt M.R., Bofetiado D.M., Cidlowski J.A. (1999). The dominant negative activity of the human glucocorticoid receptor β isoform. Specificity and mechanisms of action. J. Biol. Chem..

[64] Chatzopoulou A., Roy U., Meijer A.H., Alia A., Spaink H.P., Schaaf M.J.M. (2015). Transcriptional and metabolic effects of glucocorticoid receptor α and β signaling in zebrafish. Endocrinology.

[65] Webster K.A., Schach U., Ordaz A., Steinfeld J.S., Draper B.W., Siegfried K.R. (2017). Dmrt1 is necessary for male sexual development in zebrafish. Dev. Biol..

[66] Lin Q., Mei J., Li Z., Zhang X., Zhou L., Gui J.F. (2017). Distinct and cooperative roles of amh and dmrt1 in self-renewal and differentiation of male germ cells in zebrafish. Genetics.

[67] Hemming F.J., Aubert M.L., Dubois P.M. (1988). Differentiation of Fetal Rat Somatotropes in vitro: Effects of Cortisol, 3,5,3′-Triiodothyronine, and Glucagon, a Light Microscopic and Radioimmunological Study. Endocrinology.

[68] McCormick S.D. (1990). Cortisol directly stimulates differentiation of chloride cells in tilapia opercular membrane. Am. J. Physiol..

[69] Pereira R.M.R., Delany A.M., Durant D., Canalis E. (2002). Cortisol regulates the expression of notch in osteoblasts. J. Cell. Biochem..

[70] Sloman K.A., Desforges P.R., Gilmour K.M. (2001). Evidence for a mineralocorticoid-like receptor linked to branchial chloride cell proliferation in freshwater rainbow trout. J. Exp. Biol..

[71] Feng X., Reini S.A., Richards E., Wood C.E., Keller-Wood M. (2013). Cortisol stimulates proliferation and apoptosis in the late gestation fetal heart: Differential effects of mineralocorticoid and glucocorticoid receptors. Am. J. Physiol.-Regul. Integr. Comp. Physiol..

[72] Cruz S.A., Chao P.L., Hwang P.P. (2013). Cortisol promotes differentiation of epidermal ionocytes through Foxi3 transcription factors in zebrafish (*Danio rerio*). Comp. Biochem. Physiol.-A Mol. Integr. Physiol..

[73] Cruz S.A., Lin C.-H., Chao P.-L., Hwang P.-P. (2013). Glucocorticoid Receptor, but Not Mineralocorticoid Receptor, Mediates Cortisol Regulation of Epidermal Ionocyte Development and Ion Transport in Zebrafish (*Danio Rerio*). PLoS ONE.

[74] Li N., Oakes J.A., Storbeck K., Cunliffe V.T., Krone N. (2020). The P450 side chain cleavage enzyme Cyp11a2 facilitates steroidogenesis in zebrafish. J. Endocrinol..

[75] Tang H., Chen Y., Wang L., Yin Y., Li G., Guo Y., Liu Y., Lin H., Cheng C.H.K., Liu X. (2018). Fertility impairment with defective spermatogenesis and steroidogenesis in male zebrafish lacking androgen receptor. Biol. Reprod..

[76] de Gendt K., Swinnen J.V., Saunders P.T.K., Schoonjans L., Dewerchin M., Devos A., Tan K., Atanassova N., Claessens F., Lécureuil C. (2004). A Sertoli cell-selective knockout of the androgen receptor causes spermatogenic arrest in meiosis. Proc. Natl. Acad. Sci. USA.

[77] Morais R.D.V.S., Crespo D., Nóbrega R.H., Lemos M.S., van de Kant H.J.G., de França L.R., Male R., Bogerd J., Schulz R.W. (2017). Antagonistic regulation of spermatogonial differentiation in zebrafish (*Danio rerio*) by Igf3 and Amh. Mol. Cell. Endocrinol..

[78] Safian D., Ryane N., Bogerd J., Schulz R.W. (2018). Fsh stimulates Leydig cell Wnt5a production, enriching zebrafish type A spermatogonia. J. Endocrinol..

[79] Safian D., Bogerd J., Schulz R.W. (2019). Regulation of spermatogonial development by Fsh: The complementary roles of locally produced Igf and Wnt signaling molecules in adult zebrafish testis. Gen. Comp. Endocrinol..

[80] Adolfi M.C., Nakajima R.T., Nóbrega R.H., Schartl M. (2019). Intersex, Hermaphroditism, and Gonadal Plasticity in Vertebrates: Evolution of the Müllerian Duct and Amh/Amhr2 Signaling. Annu. Rev. Anim. Biosci..

[81] Skaar K.S., Nóbrega R.H., Magaraki A., Olsen L.C., Schulz R.W., Male R. (2011). Proteolytically Activated, Recombinant Anti-Müllerian Hormone Inhibits Androgen Secretion, Proliferation, and Differentiation of Spermatogonia in Adult Zebrafish Testis Organ Cultures. Endocrinology.

[82] Di Carlo A., Travia G., de Felici M. (2000). The meiotic specific synaptonemal complex protein SCP3 is expressed by female and male primordial germ cells of the mouse embryo. Int. J. Dev. Biol..

[83] Yano A., Suzuki K., Yoshizaki G. (2008). Flow-Cytometric Isolation of Testicular Germ Cells from Rainbow Trout (*Oncorhynchus mykiss*) Carrying the Green Fluorescent Protein Gene Driven by Trout vasa Regulatory Regions. Biol. Reprod..

[84] Carvalho C.E., Tanaka H., Iguchi N., Ventelä S., Nojima H., Nishimune Y. (2002). Molecular Cloning and Characterization of a Complementary DNA Encoding Sperm Tail Protein SHIPPO 11. Biol. Reprod..

[85] Goetz F.W., Theofan G. (1979). *In vitro* stimulation of germinal vesicle breakdown and ovulation of yellow perch (*Perca flavescens*) oocytes. Effects of 17α-hydroxy-20β-dihydroprogesterone and prostaglandins. Gen. Comp. Endocrinol..

[86] Upadhyaya N., Haider S. (1986). Germinal vesicle breakdown in oocytes of catfish, *Mystus vittatus* (Bloch): Relative *in vitro* effectiveness of estradiol-17β, androgens, corticosteroids, progesterone, and other pregnene derivatives. Gen. Comp. Endocrinol..

[87] Colombe L., Fostier A., Bury N., Pakdel F., Guiguen Y. (2000). A mineralocorticoid-like receptor in the rainbow trout, *Oncorhynchus mykiss*: Cloning and characterization of its steroid binding domain. Steroids.

[88] Bury N.R., Sturm A., Le Rouzic P., Lethimonier C., Ducouret B., Guiguen Y., Robinson-Rechavi M., Laudet V., Rafestin-Oblin M.E., Prunet P. (2003). Evidence for two distinct functional glucocorticoid receptors in teleost fish. J. Mol. Endocrinol..

[89] Prunet P., Sturm A., Milla S. (2006). Multiple corticosteroid receptors in fish: From old ideas to new concepts. Gen. Comp. Endocrinol..

[90] Levy F.O., Ree A.H., Eikvar L., Govindan M.V., Jahnsen T., Hansson V. (1989). Glucocorticoid receptors and glucocorticoid effects in rat sertoli cells. Endocrinology.

[91] Schultz R., Isola J., Parvinen M., Honkaniemi J., Wikström A.C., Gustafsson J.A., Pelto-Huikko M. (1993). Localization of the glucocorticoid receptor in testis and accessory sexual organs of male rat. Mol. Cell. Endocrinol..

[92] Biagini G., Pich E.M., Frasoldati A., Agnati L.F., Marrama P. (1995). Changes in glucocorticoid receptor immunoreactivity after adrenalectomy and corticosterone treatment in the rat testis. J. Endocrinol. Invest..

[93] Weber M.-A., Groos S., Höpfl U., Spielmann M., Aumüller G., Konrad L. (2000). Glucocorticoid receptor distribution in rat testis during postnatal development and effects of dexamethasone on immature peritubular cells in vitro. Andrologia.

[94] Hazra R., Upton D., Jimenez M., Desai R., Handelsman D.J., Allan C.M. (2014). In Vivo Actions of the Sertoli Cell Glucocorticoid Receptor. Endocrinology.

[95] Consten D., Keuning E.D., Bogerd J., Zandbergen M.A., Lambert J.G.D., Komen J., Goos H.J.T. (2002). Sex Steroids and Their Involvement in the Cortisol-Induced Inhibition of Pubertal Development in Male Common Carp, *Cyprinus carpio* L.. Biol. Reprod..

