# Supplementary material for: Cortisol Directly Stimulates Spermatogonial Differentiation, Meiosis, and Spermiogenesis in Zebrafish (Danio rerio) Testicular Explants
Source: Biomolecules. 2020 Mar 10;10(3):429. doi: 10.3390/biom10030429 (PMC7175196; doi:10.3390/biom10030429)
Supplement: Supplementary file 1 [file biomolecules-10-00429-s001.zip › Figure S1.docx]

**
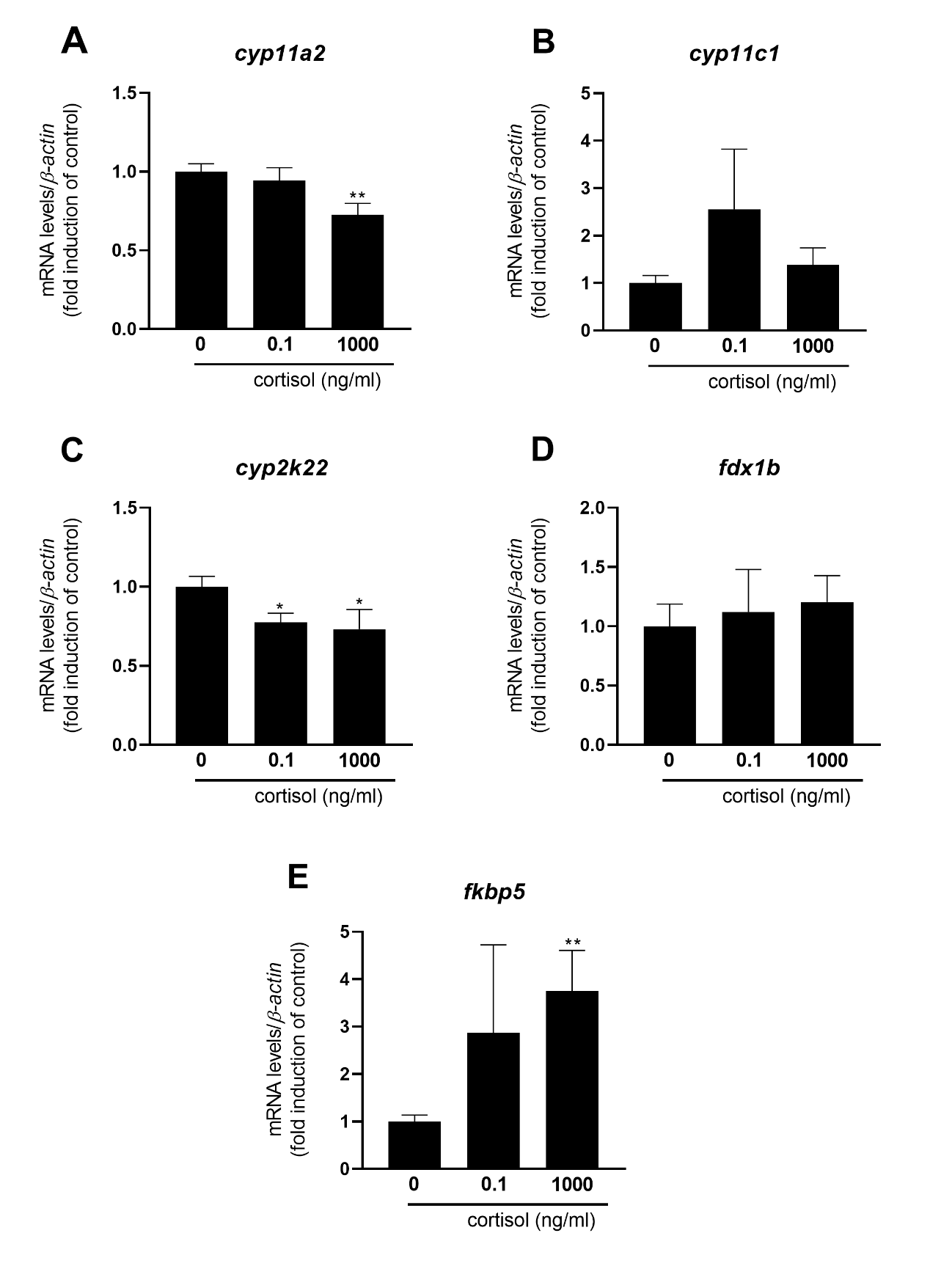
**

**Supplemental Figure 1.** Relative mRNA levels of several selected genes in zebrafish testes incubated for 18 hours (short-term exposure) to increasing concentrations of cortisol (0; 0.1 and 1000 ng/mL). The selected target genes *cyp11a2* (cytochrome P450, family 11, subfamily A, polypeptide 2) (A), *cyp11c1* (cytochrome P450, family 11, subfamily C, polypeptide 1) (B), *cyp2k22* (cytochrome P450, family 2, subfamily K, polypeptide 22) (C), *fdx1b* (ferredoxin 1b) (D), and *fkbp5* (FKBP prolyl isomerase 5) (E) were evaluated. Ct values were normalized with *β-actin* and expressed as relative values of basal (0 ng/mL) levels of expression. Bars represent the mean ± SEM fold change (n = 6), relative to the control (basal - 0 ng/mL), which is set at 1. Paired t-test, ** p<0.01; * p<0.05.
